# Harnessing Mirror Neurons: Improving Balance and Quality of Life After a Stroke

**DOI:** 10.7759/cureus.81290

**Published:** 2025-03-27

**Authors:** Preeti Sharma, Zeeshan Ali, Sudhan S George

**Affiliations:** 1 Department of Physiotherapy, Krupanidhi College of Physiotherapy, Bengaluru, IND; 2 Department of Physiology, Krupanidhi College of Physiotherapy, Bengaluru, IND

**Keywords:** action observation, balance, bbs score, mirror neuron, quality of life, ss-qol

## Abstract

Background: Action observation engages brain motor networks, and action imitation helps neurological and musculoskeletal problem patients improve motor learning and functional recovery. In this study, we focused on identifying the impact of action observation training (AOT) on balance and quality of life (QOL) in hemiparetic stroke patients.

Method: A quasi-experimental study in Bengaluru (from December 2021 to July 2022) involved 60 hemiparetic patients randomly divided into two groups. Group A received 30 minutes of AOT alongside standard physiotherapy, while Group B received conventional therapy. Sessions focused on balance exercises, and outcomes were assessed using the Berg Balance Scale (BBS) and the Stroke-Specific Quality of Life (ss-QOL) scale. Statistical analyses, including paired and independent t-tests, highlighted significant differences, ensuring methodological rigor and ethical compliance.

Results: The findings show a significant difference in the pretest BBS scores between the experimental and control groups (p = 0.010), with the experimental group having a lower baseline. However, there was no significant difference in posttest BBS scores (p = 0.431). Both groups showed significant improvements in their BBS and ss-QOL scores (p < 0.05). The experimental group showed a larger improvement in BBS (p = 0.001), while ss-QOL improvements were not statistically significant (p = 0.732).

Conclusion: The study concluded that the experimental and control groups demonstrated significant improvements in balance and QOL after the intervention, with the experimental group showing significantly larger improvements in balance, compared to control group.

## Introduction

Stroke is the major cause of adult disability and also a predominant reason for mortality and morbidity in developed countries and is a notable global health problem [1]. It is advancing in low-middle-income countries where the burden is above and beyond that of high-income countries [2]. Per annum, 15 million people suffer from a stroke at a global level. Out of all this, five million die, and another five million continue to live with a remained permanent disability, being a burden on family and community. The remaining five million people from the 15 million who suffer an episode of stroke each year are those who survive but recover to some degree, either fully or with some level of impairment that is not permanent [3]. In India, stroke’s prevalence rate is anticipated to be 84-262 out of 100,000 people in rural areas and 334-424 out of 100,000 people in city locations [4]. Based on the latest population-based studies, the incidence rate has come out to be 119-145 in 100,000 people [4]. This leads to motor impairments and functional disabilities, followed by a stroke [1].

Stroke is defined as "rapidly developed clinical signs of focal (or global) disturbance of cerebral function, lasting more than 24 hours or leading to death, with no apparent cause other than of vascular origin," as stated by the World Health Organization in 1970 [5]. The commonest category of it is ischemic stroke, impacting about 80% of individuals. When the brain is deprived of vital oxygen and nutrients, it leads to impaired blood flow or a clot blockage. Although ischemic stroke is relatively common among all stroke cases, hemorrhagic stroke still contributes significantly to the global burden of disability-adjusted life-years [6,7].

Stroke is the main reason for disability, which compromises ambulation and a person’s ability to execute daily living activities [1]. One of the most common deficits following a stroke is lower limb impairments and postural imbalance, which drastically impact physical ability [8]. The neurological deficit, which is one of the most frequent after stroke, is hemiparesis [9]. Anterior cerebral artery strokes often result in contralateral hemiparesis and sensory loss, with a greater impact on the lower extremity than the upper extremity (UE). Hemiparesis is a leading cause of long-term disability, affecting mobility in 50% of stroke survivors and leaving 26% unable to perform basic daily activities independently. Approximately 80% of stroke patients exhibit some degree of contralateral hemiparesis, significantly affecting their health-related quality of life (HRQOL) and ability to engage in personal and domestic activities of daily living (ADLs). These challenges often lead to declines in mental health, well-being, and overall quality of life (QOL) [10-12].

Within six months after stroke, 73% of patients experience falls due to balance impairments linked to sensory, cognitive, motor, or integrative movement control deficits. Additional complications such as muscle weakness, sensory deficits, cognitive impairments, joint range of motion (ROM) limitations, altered muscle tone, and abnormal postural reactions exacerbate balance issues [13-16].

The Berg Balance Scale (BBS) is widely recognized for assessing balance in acute stroke patients, offering a standardized tool for neurological rehabilitation practitioners. For measuring HRQOL, the Stroke-Specific Quality of Life (ss-QOL) scale is validated across diverse stroke populations, making it a valuable measure for this study [17-20].

Rehabilitation strategies aim to improve motor relearning through neuroplasticity-focused approaches, such as constraint-induced movement therapy, robot-assisted therapy, and virtual reality-based rehabilitation [21-23]. These techniques work intensively to retrain motor functions in the affected hemispheres. However, overcoming the "learned nonuse" phenomenon and enhancing ipsilateral motor cortex function remain essential for severe hemiparesis cases [24-26].

Functional improvement, including balance, posture symmetry, and weight-shifting abilities, is critical for enabling stroke patients to perform ADLs effectively and enhance social participation [27-30]. Innovative therapies like action observation training (AOT) leverage the mirror neuron system (MNS) to activate motor-related cortical regions through goal-directed activity observation followed by replication [31-35]. This activation facilitates the strengthening of intact cortical networks and the repair of damaged ones, preventing cortical deterioration in immobile patients.

Our study hypothesizes that AOT may provide superior outcomes in enhancing balance and QOL for hemiparetic stroke patients, emphasizing the potential of action observation followed by execution over passive observation [36-39].

## Materials and methods

Research design

A quasi-experimental study was conducted in and around Bengaluru, India, from December 2021 to July 2022. In total, 60 patients with hemiparesis participated in the present investigation. After getting approved by the Institutional Ethical Committee of Krupanidhi College of Physiotherapy in affiliation with Rajiv Gandhi University of Health Sciences, Bengaluru, on August 17, 2021 (ref no. EC-MPT/21/PHY/D13), informed consent was obtained. The selected population was divided into two groups that were randomly allocated at the beginning of the study to minimize the selection bias. Participants were selected based on the following criteria. The patients with a stroke diagnosis with a mini-mental state examination score greater than 24 months after stroke, a disability level assessed by the BBS with scores between 46 and 65 years, and the ischemic stroke subtype classified as subacute hemiparetic stroke were included. This selection ensured a homogeneous group for baseline assessment comparisons.

Hemorrhagic stroke, unilateral neglect, contractures of the lower limb, epilepsy, and other neurological diseases or the visual or auditory deficit that could have interfered with the study, and issues related to balance due to any neurological or musculoskeletal disorders were excluded from the study.

Allocation

**Figure 1:**
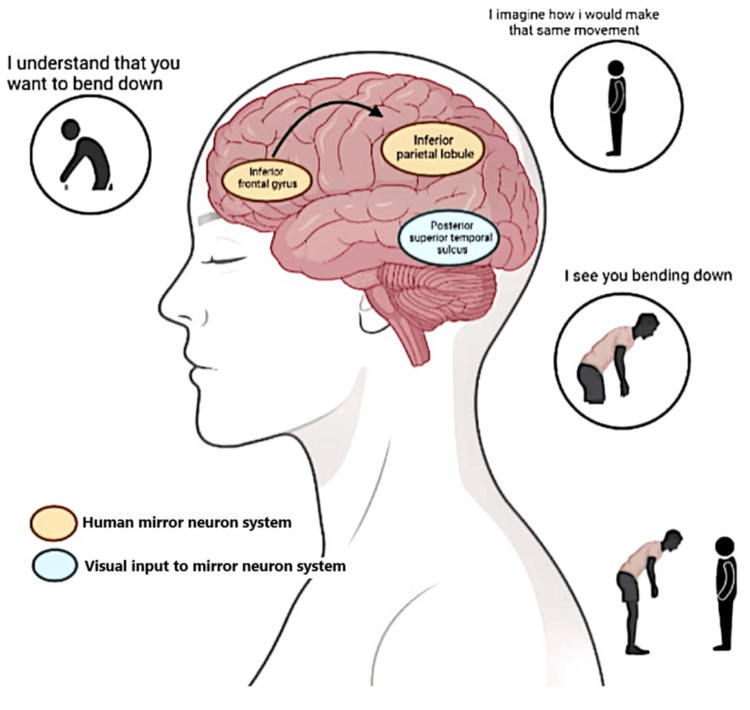
Action observation training Image credit: This is an original image created by the author Preeti Sharma

Using a computer-generated randomization scheme, participants were randomly assigned to either the active or control group. The allocation was concealed to prevent selection bias. Random allocation was made to either the experiment or the control group. The experiment group received standard AOT, as represented in Figure 1, for 15 minutes, followed by an action execution session of 15 minutes. Throughout the rehabilitation session, subjects were asked to observe the specific balance actions presented in a video displayed on a computer screen, and later, they were asked to perform the observed action. In one rehab session, just one exercise was practiced. In the video, actions were produced from all the aspects (front, back, and lateral sides) separately for hemiparetic patients (both right and left-sided) to avoid any deviation.

Intervention

The subjects had to sit comfortably in an armchair during the video presentation. They were asked to focus on videos of motion performed by other people through the monitor at a distance of 1 m. After observing the motor act (observation phase), subjects were required to simulate the watched action (execution phase). The patient was required to execute the observed action to the best of their abilities. Nevertheless, they were advised that the emphasis is on the observing component of the move, not its implementation. The treatment was done thrice a week for eight weeks. The basic level balance exercises seemed simple at first, but a strong neural connection was required to complete them.

The first stage (sitting movement) focuses on improving balance in a seated position by shifting weight forward, side to side, rotating left and right, and performing weight shifts on a Swiss ball. The second stage (sit-to-stand movement) involves pelvis adjustments in a seated position, weight shifts forward, and standing up from a sitting position. The third stage (standing movement) includes exercises to enhance balance, such as heel raises (with support), sidestepping (with support), heel raises (without support), sidestepping (without support), heel-to-toe walking, single-leg standing, and single-leg standing with eyes closed. The final fourth stage (sideward walking) includes walking to the left and right, backward walking, turning 180°, and navigating obstacles while walking.

Group B was a control group receiving conventional physiotherapy treatment, which was openly endorsed by the patient’s neurologist or rehabilitation physician, according to medical opinion and patient requests. In this study, physiotherapy was freely chosen by the patient or prescribed by the physician as part of a repetitive practice regimen. The control group received 60 minutes of standard physical therapy, while the active group participated in 30 minutes of standard therapy followed by 30 minutes of additional AOT. This ensures that the only difference between the groups was the added component of AOT in the experimental group. To address this concern, we have revised the study design such that both groups receive 60 minutes of standard physical therapy, with the active group also receiving video observation for mirror neuron activation through imitation.

Routine physiotherapy includes active ROM, passive ROM, active assistive ROM, stretching, resistance training, balance and coordination activities, and large-muscle movements, including walking, using a treadmill, and a stationary cycle, both while seated and standing. The program was delivered thrice a week for eight weeks.

During the trial period, all individuals were told to participate in accordance with the contents of their respective programs. On the first day and eighth week of training, the BBS and ss-QOL were administered to all participants to assess their balance and HR-QOL. The examinations were administered by the same examiner using the same testing procedures.

Statistical analysis

Data were evaluated using IBM Statistical Package for the Social Sciences 2019, IBM Corporation, Chicago, IL, for Windows. Demographic data and outcome variables were calculated using descriptive statistics. The BBS and ss-QOL were utilized for hemiparetic stroke patients to determine significant differences between the variables using a paired t-test. An independent t-test determined the statistical significance of the difference between the groups. MS Excel software (Microsoft Corporation, Redmond, WA) was used to create graphs and tables. The null hypothesis is that there is no significant improvement in the balance or QOL after the intervention. The alternate hypothesis is that there is a significant improvement in the balance or QOL.

All participants in both groups received standard physical therapy as part of their rehabilitation plan, and the institutional review board approved the study. The ethical implications of additional AOT in the active group were carefully considered, and participants gave informed consent to the additional intervention.

## Results

The research analyzed 60 hemiparetic stroke patients who met the inclusion and exclusion criteria. The baseline characteristics (Table 1) of the experimental and control groups were compared across four variables: age, weight, height, and BMI. The experimental group had a mean age of 52.07 ± 6.175 years, while the control group had a mean age of 54.80 ± 8.422 years, with the control group being slightly older on average. In terms of weight, the experimental group had a mean weight of 61.80 ± 10.344 kg, which was significantly lower than the control group's mean weight of 71.53 ± 9.250 kg. This indicates that the control group had a higher average weight. For height, the experimental group had a mean height of 161.97 ± 10.384 cm, while the control group had a mean height of 165.83 ± 6.265 cm, showing that the control group was slightly taller on average. Finally, regarding BMI, the experimental group had a mean BMI of 23.43 ± 2.24, while the control group had a higher mean BMI of 26.31 ± 2.18, suggesting that the control group had a higher average BMI. These data reflect notable differences between the two groups in weight, height, and BMI, which may be relevant to further analysis, particularly when considering the potential impact of these variables on the study's outcomes.

**Table 1:** Mean and standard deviation of baseline characteristics of the experimental and control groups

Variables	Experimental group		Control group		p value
	Mean	Standard deviation	Mean	Standard deviation	
Age (years)	52.07	6.175	54.8	8.422	0.685
Weight (kg)	61.8	10.344	71.53	9.25	0.754
Height (m)	161.97	10.384	165.83	6.265	0.85
BMI	23.4327	2.23823	26.3067	2.17968	0.945

**Figure 2:**
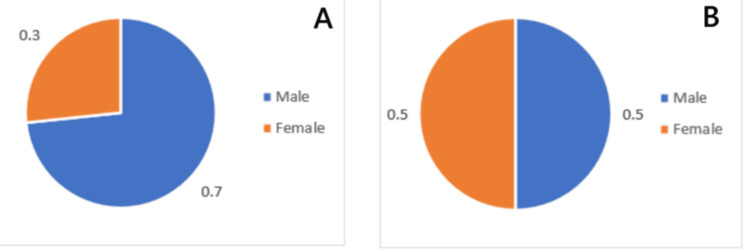
Gender distribution of the (A) experimental and (B) control groups

Figure 2 presents data on gender distribution. Of the total respondents, 22 are male, accounting for 73.3% of the total sample, while eight are female, making up 26.7%. This indicates a significantly higher proportion of males than females in the experimental group.

Table 2 compares the mean and standard deviation (SD) of the BBS scores for both the experimental and control groups at two time points: Berg Balance Scale pretreatment (BBS PRE) and Berg Balance Scale posttreatment (BBS POST). For the pretreatment scores, the experimental group had a mean of 37.40 ± 6.584, indicating a relatively lower balance ability with some variability. In contrast, the control group had a mean of 41.00 ± 3.270, suggesting a higher baseline balance ability with less variability. After the intervention, the experimental group showed improvement, with a mean posttreatment score of 45.53 ± 5.342, indicating a moderate increase in balance ability, although with some variability among participants. The control group also showed improvement, with a posttreatment mean of 46.47 ± 3.598, reflecting a higher posttreatment score than the experimental group but with a similar level of consistency. Overall, both groups demonstrated improvements in balance following the intervention, but the control group maintained higher scores at both time points. The experimental group exhibited a larger improvement in balance, albeit with more variability in the results.

**Table 2:** Findings of the BBS and ss-QOL scale of the experimental and control groups BBS PRE: Berg Balance Scale preintervention; BBS POST: Berg Balance Scale postintervention; ss-QOL PRE: Stroke-Specific Quality of Life Scale preintervention; ss-QOL POST: Stroke-Specific Quality of Life Scale postintervention

Variables	Experimental group		Control group		p value
	Mean	Standard deviation	Mean	Standard deviation	
BBS PRE	37.4	6.584	41	3.27	0.01
BBS POST	45.53	5.342	46.47	3.598	0.431
ss-QOL PRE	143.47	30.475	155.47	22.122	0.087
ss-QOL POST	194.1	24.873	192	22.297	0.732

At baseline, the experimental group had a mean ss-QOL score of 143.47 with an SD of 30.475, indicating a relatively lower QOL score with considerable variability. The control group, on the other hand, had a higher mean pretreatment score of 155.47 with an SD of 22.122, suggesting better baseline QOL and less variability in scores. After the intervention, both groups showed improvements in their ss-QOL scores. The experimental group had a posttreatment mean of 194.10 with an SD of 24.873, indicating a significant improvement in QOL, with moderate variability among participants. The control group also demonstrated an increase, with a posttreatment mean of 192.00 and an SD of 22.297, reflecting an improvement, though slightly lower than that of the experimental group.

**Figure 3:**
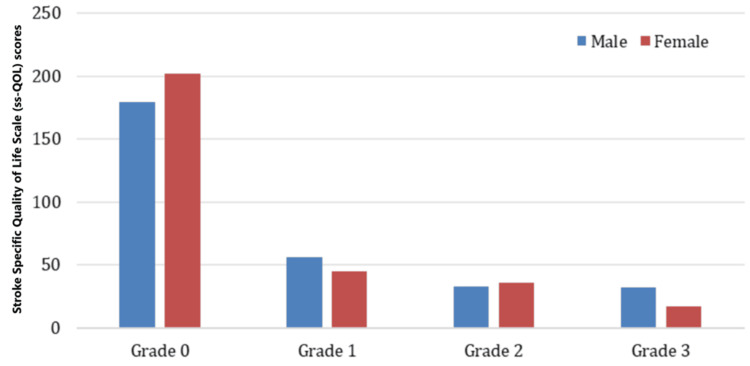
Mean and standard deviation of Stroke-Specific Quality of Life scale of experimental and control group

Figure 3 shows the mean and SD of the ss-QOL scale scores categorized by gender across different grades (Grades 0-3). For Grade 0, both men and women exhibit the highest mean scores compared to the other grades, with women slightly exceeding men in this category. This indicates that individuals in Grade 0, regardless of gender, tend to have a higher QOL, with women showing a marginal advantage. In Grade 1, the scores for both genders decrease, but males show a slightly higher mean score than women. As we move to Grade 2, the trend continues with further reductions in mean scores, and both genders show similar values, reflecting minimal gender differences in this grade. For Grade 3, the scores are the lowest among all categories, with men exhibiting a slightly higher mean score than women.

A paired t-test (Table 3) analyzed experimental group pre- and posttests. For the BBS in the experimental group, there was a significant difference in mean of -8.133, SD of 3.803, t value of -11.713, and p value of 0.001. By using the student t-distribution table, the p value was found to be less than the threshold chosen for significance, which led to the acceptance of an alternate hypothesis for the balance. For the ss-QOL score, a significant difference was seen with a mean value of -50.633, SD of 24.374, t value of -11.378, and p value of 0.000. p value calculated using the student t-distribution table came out to be less than the threshold selected for significance; hence, we accept an alternate hypothesis for ss-QOL. A paired t-test analyzed the control group pre- and posttests. For BBS, a significant difference in the control group with a mean of -5.467, SD of 2.270, t value of -13.189, and p value of 0.000 was observed. p value was found using the student t-distribution table, and the calculated p value was less than the criterion significance value (p < 0.05); hence, we accept an alternate hypothesis for the balance scale (Table 4).

**Table 3:** Results from the experimental and control groups using paired t-test *p < 0.05 BBS: Berg Balance Scale; ss-QOL: Stroke-Specific Quality of Life

Tests	Mean	Standard deviation	t-value	p value
Experiment group				
BBS pretest score: BBS posttest score	-8.133	3.803	-11.713	0.001^*^
ss-QOL pretest score: ss-QOL posttest score	-50.633	24.374	-11.378	0.001^*^
Control group				
BBS pretest score: BBS posttest score	-5.467	2.27	-13.189	0.000^*^
ss-QOL pretest score: ss-QOL posttest score	-36.533	17.612	-11.362	0.000^*^

**Table 4:** Results from the experimental and control groups using independent t-test BBS: Berg Balance Scale; ss-QOL: Stroke-Specific Quality of Life ^*^Statistically significant **Statistically nonsignificant

Variables	Groups	Mean	Standard deviation	t-value	p value
BBS pretest score	Group A (Experimental)	37.4	6.584	-2.682	0.010^*^
	Group B (Control)	41	3.27		
BBS posttest score	Group A (Experimental)	45.53	5.342	-0.794	0.431^**^
	Group B (Control)	46.47	3.598		
ss-QOL pretest score	Group A (Experimental)	143.47	30.475	-1.745	0.086^**^
	Group B (Control)	155.47	22.122		
ss-QOL posttest score	Group A (Experimental)	194.1	24.873	0.344	0.732^**^
	Group B (Control)	192	22.297		

An independent t-test (Table 4) was done for the analysis between an experimental group and a control group. For the pretest BBS score, there was a significant difference with a t = -2.682 and a p = 0.010, leading to the acceptance of the alternate hypothesis. For the posttest BBS score, there was no significant difference with t = -0.794 and p = 0.431, indicating our results in favor of the null hypothesis.

## Discussion

An improvement in static and dynamic balance is thought to be crucial for improving social engagement and HR-QOL in chronic stroke patients [40]. No matter if the activity had been memorized or forgotten, individuals replicated the activity after seeing the videos again as part of AOT. The steps in this action imitation process include action observation, motor imagery, and action execution [41]. The discovery of the MNS provides a solid physiological foundation for this strategy [42]. It is thought that action observation facilitates detection and error correction and additionally works on motor imagery when learning takes place [43]. Hemiparetic patients ameliorate their static and dynamic balance of the part that is not functional by imitating the action through action observation [41].

The objective of the study is to determine how AOT, which is based on the MNS, affects balance and QOL in people who have had hemiparetic strokes. The control group underwent conventional physiotherapy treatment that was freely prescribed by the patient-attending clinician, neurologist, or rehab physician based on medical opinion and patient wishes, while the experimental group watched the actions and imitated the observed activities. There has been an improvement in motor learning tasks involving the UEs, according to several earlier studies on the subject [43]. The study's findings revealed that the action observation group demonstrated analogous degrees of enhancement in BBS and ss-QOL scores.

A study on actual training and motor imaging exhibited improvements in terms of functional activity, which supports the findings of the present study [41]. Another study showed that the exercise therapy treatment along with action observation, where participants repeatedly performed the observed activity, may show advancements in the function of brains that have been damaged [44].

Studies reported that when viewed using functional magnetic resonance imaging, individuals who received AOT exhibited a strong signal in the afflicted primary and supplemental motor regions [37]. Similar AOT studies have shown that patients who watched videos of daily life before receiving conventional treatment therapy experienced greater improvements in terms of motor function, muscle spasticity, and daily living activities after four weeks of treatment [45,46].

Similar to the earlier research described, patients' balance and QOL fundamentals improved after they attended AOT. Considering the outcomes of this research, it can be inferred that patients' motor projection areas were activated when observing the performed actions. This suggests that action observation activates neural networks in the cerebellum and cerebrum of hemiparetic patients, which results in brain reorganization and helps to improve balance skills [43].

During all phases of stroke recovery, improving the patient's poor balance is more important than teaching them compensatory techniques. Studies reported important relationships between balance and QOL in chronic stroke patients. It was discovered that numerous factors might either negatively or favorably impact the QOL in poststroke hemiparetic individuals. According to this study, balance is the main indicator of physical recovery [42,43]. Lower degrees of physical impairment and increasing disability are linked to higher levels of balance impairment, which accounts for lower QOL. Hence, according to the results of this study, an important relationship exists between balance and QOL in hemiparetic stroke patients. Therefore, improved balance impairments resulted in the enhancement of QOL in this study population [42-44].

It is widely documented that AOT stimulates parts of the motor network and MNS, such as ventral IPL, premotor cortex, and inferior frontal gyrus, while acquiring new motor skills and when seeing behaviors that are already part of the observer's motor repertoire. MNS is essential for motor learning, which is defined as "a collection of mechanisms linked with practice that result in relatively permanent changes in the capacity to move" [47]. The results indicate that AOT is a useful technique that may be applied in clinical settings because it is made up of procedures that involve observing movement task videos and putting them into practice, making it simple for stroke patients who are motivated to recover to apply. Because patients may continue this training via videos even after being discharged from the hospital, it can also benefit cost efficiency [48]. When used in conjunction with conventional treatment techniques as a pretraining procedure before training in stroke rehabilitation to improve abilities, AOT has been demonstrated to be more successful. However, this technique may only be put into practice when patients are highly motivated and capable of concentrating.

The results show that stroke patients can benefit from using the mirror neurons theory in their rehabilitation therapy. The MNS was engaged by observation and imitation, which might encourage the patients to learn new motor abilities. By watching the forthcoming training movement, mirror neurons that regulate the identical motion in stroke patients with hemiparesis may be stimulated, and their excitability might be raised. As a result, the capacity of mirror neurons to complete the training was enhanced. Therefore, imitation learning can enhance motor skills.

In addition to the suggested neurological mechanism underpinning the benefit of action observation, it was discovered that the patient's desire and zeal for rehabilitation had increased. Hence, this therapy has helped accelerate the dynamic and static balance impairments in hemiparetic stroke patients, thereby effectively improving the efficacy of the treatment. As a result, using this rehabilitation approach has been beneficial in enhancing prognosis and QOL in our study subjects.

Thus, these shreds of evidence indicate that the AOT has a favorable effect on balance deficits and, consequently, improves the QOL in hemiparetic stroke patients, expanding the scope of AOT’s application and offering a successful therapeutic approach for hemiparesis patients. According to this proposed treatment regimen, the MNS functions as a specific physiological motor network, implying that the improvement depends on the reactivation of motor representations associated with the observed activities. It is advised that significant efforts be made to enhance the balance and QOL of stroke and other neurologic patients by creating more varied AOT approaches. This study has some limitations that can be improved by further research. This study was done for two months. Also, the daily activities of the subject were not monitored, which could have influenced the study. Hence, to identify how to successfully apply AOT in clinical practice, more studies with a longer follow-up and relations with instrumental data are recommended.

## Conclusions

The study concluded that there is a significant improvement in both balance and QOL for hemiparetic stroke patients who received AOT. The group that received AOT showed a more considerable improvement in balance, although the group that did not receive AOT maintained higher baseline balance scores. The AOT group also showed a larger improvement in their stroke-specific QOL, though both groups experienced positive changes posttreatment. Statistical analyses highlighted significant differences in pre- and posttreatment scores, particularly for the BBS and stroke-specific QOL, indicating the effectiveness of AOT. However, there were no significant differences in posttreatment scores between the two groups, suggesting that AOT had a comparable beneficial effect on both. The male-dominant gender distribution in the sample may limit the generalizability of the results. Additionally, the observed differences in weight, height, and BMI across participants may have influenced the outcomes and should be considered for further analysis.
